# Effect of spatiotemporal variables on abundance, biting activity and parity of *Nyssorhynchus darlingi* (Diptera: Culicidae) in peri-Iquitos, Peru

**DOI:** 10.1186/s12936-024-04940-z

**Published:** 2024-04-19

**Authors:** Sara A. Bickersmith, Marlon P. Saavedra, Catharine Prussing, Rachel E. Lange, Juliana A. Morales, Freddy Alava, Joseph M. Vinetz, Dionicia Gamboa, Marta Moreno, Jan E. Conn

**Affiliations:** 1 Wadsworth Center, New York State Department of Health, Albany, NY USA; 2 Laboratorio ICEMR–Amazonia, Laboratorios de Investigación y Desarrollo, Facultad de Ciencias E Ingeniería, Universidad Peruana Cayetano Heredia, Lima, Peru; 3 Department of Biomedical Sciences, School of Public Health, State University of New York-Albany, Albany, NY USA; 4 Gerencia Regional de Salud de Loreto (GERESA), Iquitos, Peru; 5 Section of Infectious Diseases, Department of Internal Medicine, Yale School of Medicine, New Haven, CT USA; 6 Instituto de Medicina Tropical “Alexander Von Humboldt”, Universidad Peruana Cayetano Heredia, Lima, Peru; 7 Departamento de Ciencias Celulares y Moleculares, Facultad de Ciencias e Ingeniería, Universidad Peruana Cayetano Heredia, Lima, Peru; 8 Department of Infection Biology, London School of Hygiene & Tropical Medicine, London, UK

**Keywords:** *Nyssorhynchus darlingi*, Amazonian Peru, Malaria transmission, Vectors, Abundance, Seasonality

## Abstract

**Background:**

In malaria endemic regions of the Peruvian Amazon, rainfall together with river level and breeding site availability drive fluctuating vector mosquito abundance and human malaria cases, leading to temporal heterogeneity. The main variables influencing spatial transmission include location of communities, mosquito behaviour, land use/land cover, and human ecology/behaviour. The main objective was to evaluate seasonal and microgeographic biting behaviour of the malaria vector *Nyssorhynchus* (or *Anopheles*) *darlingi* in Amazonian Peru and to investigate effects of seasonality on malaria transmission.

**Methods:**

We captured mosquitoes from 18:00 to 06:00 h using Human Landing Catch in two riverine (Lupuna, Santa Emilia) and two highway (El Triunfo, Nuevo Horizonte) communities indoors and outdoors from 8 houses per community, during the dry and rainy seasons from February 2016 to January 2017. We then estimated parity rate, daily survival and age of a portion of each collection of *Ny. darlingi*. All collected specimens of *Ny. darlingi* were tested for the presence of *Plasmodium vivax* or *Plasmodium falciparum* sporozoites using real-time PCR targeting the small subunit of the 18S rRNA.

**Results:**

Abundance of *Ny. darlingi* varied across village, season, and biting behaviour (indoor vs outdoor), and was highly significant between rainy and dry seasons (*p* < 0.0001). Biting patterns differed, although not significantly, and persisted regardless of season, with peaks in highway communities at ~ 20:00 h in contrast to biting throughout the night (i.e., 18:00–06:00) in riverine communities. Of 3721 *Ny. darlingi* tested for *Plasmodium*, 23 (0.62%) were infected. We detected *Plasmodium*-infected *Ny. darlingi* in both community types and most (20/23) were captured outdoors during the rainy season; 17/23 before midnight. Seventeen *Ny. darlingi* were infected with *P. vivax*, and 6 with *P. falciparum*. No infected *Ny. darlingi* were captured during the dry season. Significantly higher rates of parity were detected in *Ny. darlingi* during the rainy season (average 64.69%) *versus* the dry season (average 36.91%) and by community, Lupuna, a riverine village, had the highest proportion of parous to nulliparous females during the rainy season.

**Conclusions:**

These data add a seasonal dimension to malaria transmission in peri-Iquitos, providing more evidence that, at least locally, the greatest risk of malaria transmission is outdoors during the rainy season mainly before midnight, irrespective of whether the community was located adjacent to the highway or along the river.

**Supplementary Information:**

The online version contains supplementary material available at 10.1186/s12936-024-04940-z.

## Background

Malaria remains a pressing global health issue despite numerous intervention and treatment efforts, i.e., following a decade-long decrease, the World Health Organization (WHO) reported a worldwide increase of 8 million cases between 2015 and 2017, with an annual death toll estimated at 450,000 persons [1–3]. Overall, in the Americas, Peru contributes annually about 15% of cases [3], and within Peru, Loreto Department in northeastern Amazonian Peru accounts for most (90%) of the country-wide cases [4].

*Nyssorhynchus darlingi* (also known as *Anopheles darlingi* ([5]) is the main vector of *Plasmodium* transmission in and around the capital of Loreto, Iquitos, since its initial detection there [6] at the beginning of a major malaria epidemic [7]. This mosquito species subsequently spread extensively, likely along river systems [8, 9] through many parts of Amazonian Peru. Transmission hotspots occur mainly in the extensive network of riverine villages and along the Iquitos-Nauta highway, linked frequently to occupation (logging, charcoal production, agriculture, fish farming) [10–12]. Understanding malaria transmission in such diverse community types could help elimination efforts and reduction of the overall malaria case burden in Peru.

*Nyssorhynchus darlingi* is a dominant malaria vector because of its anthropophily, behavioural plasticity [13, 14] and preference for secondary forest and forest fragment edges [15, 16]. Frequently collected biting outdoors in many Peruvian communities [17–19] in some villages in Colombia and Brazil it bites frequently indoors [20, 21]. In general malaria vector biting location (indoors, outdoors) and time, can be modified by the use of IRS (insecticide residual spray) and/or LLINs (long-lasting insecticide nets) [14, 22, 23]. Access to a human bloodmeal [18], and environmental factors such as local temperature and humidity, also influence vector biting behaviour. The average infectivity rate of *Ny. darlingi* in Peru ranges from 0.1 to 4% [17, 18, 24], but throughout the Amazon Basin elevated human biting rates (HBR) can contribute to higher-than-expected vectorial capacity [25, 26]. Feeding on humans is common but bloodmeal analyses have demonstrated that hosts can include chickens, dogs and cattle, depending on local availability and accessibility [18, 21]. Biting in Peru occurs mainly between dusk and midnight with reported unimodal and bimodal peaks [9, 14]. The Global Fund Malaria Project PAMAFRO programme [27] was introduced in the Loreto Region, Amazonian Peru, in 2005 for 5 years to reduce malaria transmission. The primary interventions were strengthening of malaria diagnosis and detection, improved malaria case-management, use of insecticide-treated nets (ITNs) and encouragement of community participation in environmental management [28, 29]. The main outcome, by 2010, was the reduction in annual incidence rates from 48.9 to 11.6/1000 [28]. Studies in Loreto prior to the 2005–2010 PAMAFRO initiative detected near-equal proportions of *Ny. darlingi* biting indoors and outdoors [24], but since the end of the PAMAFRO programme, a long-lasting insecticidal net (LLIN) distribution campaign by the Ministry of Health, and the Malaria Cero Programme (MCP) initiated in 2017 [30], several communities have shown increased outdoor-biting [17, 19]. However, in localities where insecticide pressure was relaxed, *Ny. darlingi* again began to bite indoors frequently [14]. This shift to increased indoor biting appears to have resulted from behavioural plasticity, and perhaps also aging of LLINs, as opposed to hypothesized genetic differences in *Ny. darlingi* populations in Loreto [9, 14].

The environment is a powerful driver of mosquito-borne disease prevalence [31, 32]. Throughout much of the Amazon, the greatest risk of malaria transmission is during the rainy season [15, 17, 33], or during the wet-dry transition period [34]. Across the Brazilian Amazon, the length of the rainy season, as well as a range of socioeconomic factors, contribute the most to malaria risk [35]. This finding is consistent with earlier observations that rainfall may predict vector abundance [34], although a study in French Guiana determined that relationships between *Ny. darlingi* densities, malaria incidence, rainfall and water level were quite variable, depending on local land-cover and availability of suitable breeding habitat [36]. In Amazonian Peru, rainfall leads to an estimated 10 m increase in river levels and contributes to the generation of larval habitats [15, 37]. Seasonal abundance makes studying vector ecology and behaviour in the dry season difficult as numbers of specimens collected are frequently very low; malaria incidence is also reduced [11].

*Nyssorhynchus darlingi* is highly adapted to anthropogenic landscapes. In Brazil, deforestation patches of ~ 5 km^2^ were significantly correlated with malaria prevalence [16], partly due to this species’ preference for forest fringe habitat [38, 39]. Along the Iquitos-Nauta highway in Peru, Vittor et al*.* [15] found a 278-fold increase in the Human Biting Rate (HBR) and greater numbers of larval habitats in sites with high deforestation. Despite higher forest coverage in riverine communities compared to those located along highways, Lainhart et al*.* [9] detected a 3.33-fold higher rate of *Plasmodium* transmission in riverine communities, in addition to higher rates of HBR, infection rates (IR), and entomological inoculation rates (EIR). Understanding habitat-specific influences on *Ny. darlingi* behaviour in anthropogenic landscapes can help predict areas of greatest local risk of *Plasmodium* transmission.

This study aimed to understand the environmental effects of community location and seasonality on mosquito abundance, biting behaviour, and entomological indices linked to malaria transmission of the primary malaria vector, *Ny*. *darlingi*, in Loreto Department south of Iquitos, Peru. Unlike previous temporal studies of *Ny. darlingi* abundance [9, 14, 17] we were able to include dry season data in addition to the more common rainy season findings from four communities.

## Methods

### Study sites

**Fig. 1:**
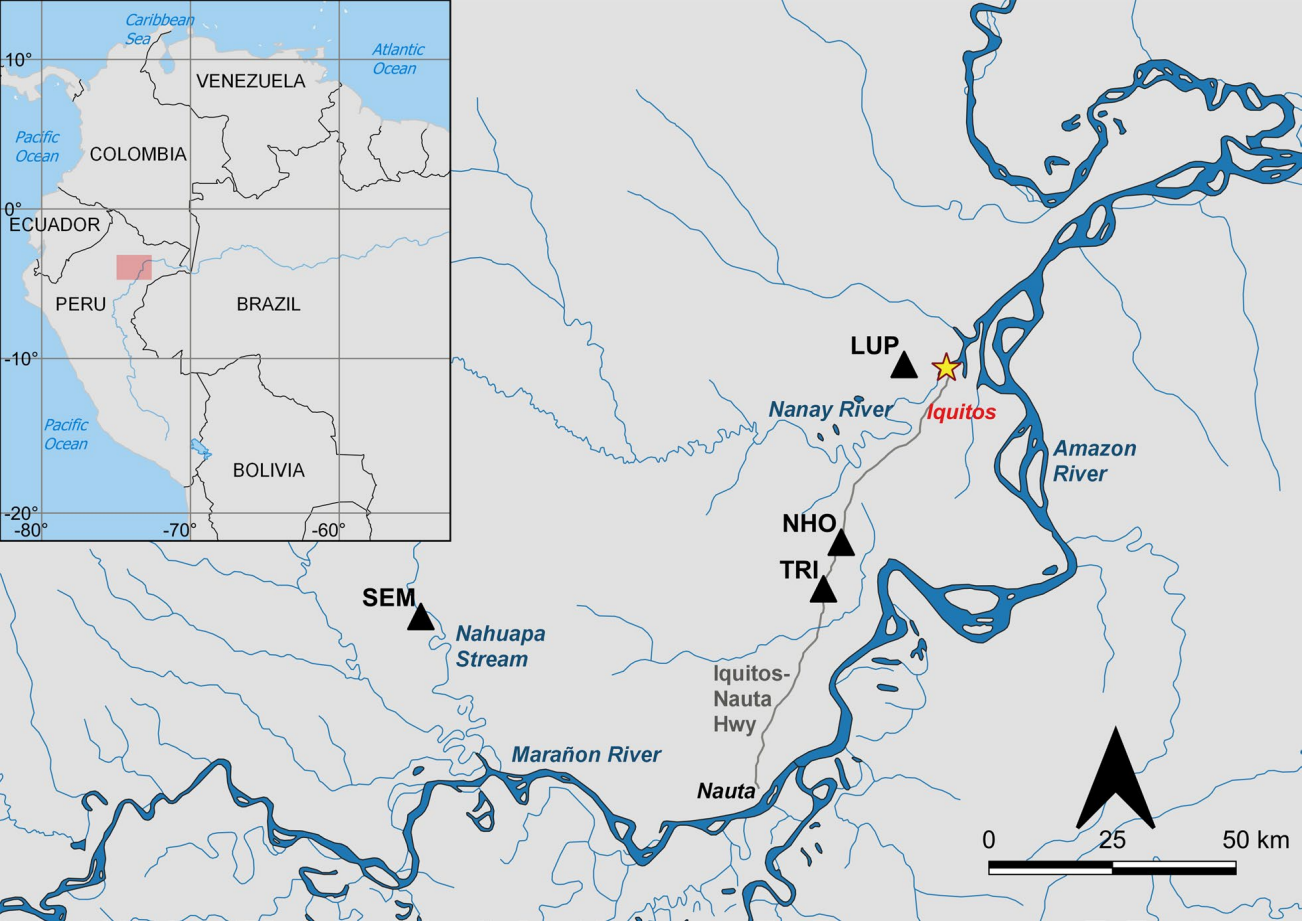
Map of communities (LUP: Lupuna, SEM: Santa Emilia, NHO: Nuevo Horizonte and TRI: El Triunfo) within Loreto Department, Peru, where *Ny. darlingi* were collected for this study. Red rectangle indicates the enlarged Loreto region of Peru. Made with Natural Earth data in QGIS v3

Adult mosquito collections were conducted in four communities in Loreto Department southwest of Iquitos (3.74°S, 73.25°W) that were each visited six times (except for El Triunfo that was visited seven times), in 2016–2017. Depending on locality, 3–4 collections were undertaken during the rainy season and 2–3 during the dry season (Table 1). Two communities studied are along rivers (Lupuna and Santa Emilia) and two along the Iquitos-Nauta highway (Nuevo Horizonte and El Triunfo) (Fig. 1). These sites have been described elsewhere: Lupuna (LUP) by Moreno et al*.* [17], Santa Emilia (SEM) by Prussing et al*.* [14], and Nuevo Horizonte (NHO) and El Triunfo (TRI) by Lainhart et al*.* [9]. As in Lainhart et al*.* [9], community habitat assignment was determined by proximity of the settlement to the nearest river: riverine localities were < 1 km and highway localities > 2 km from the nearest river.

**Table 1:** Monthly abundance, HBR and EIR of *Nyssorhynchus darlingi* captured indoor and outdoor in Lupuna (LUP), Santa Emilia (SEM), El Triunfo (TRI), and Nuevo Horizonte (NHO), 2016–2017

		Indoor			Outdoor		
Community	Mo-Yr	N	HBR(± SE)	EIR	N	HBR(± SE)	EIR
LUP	*Feb-16*	*164*	*20.5(1)*	*0.000*	*350*	*43.8(5.4)*	*0.090*
	*Apr-16*	*154*	*19.2(3.3)*	*0.000*	*328*	*41(9.1)*	*0.088*
	Jul-16	95	11.9(0.5)	0.000	192	24(4.9)	0.000
	Oct-16	34	4.2(1)	0.000	57	7.1(0.9)	0.000
	Dec-16	27	3.8(0.5)	0.000	43	5.4(0.7)	0.000
	*Jan-17*	*172*	*21.5(1.5)*	*0.000*	*273*	*34.1(3.4)*	*0.000*
	Subtotal	646			1243		
SEM	*Feb-16*	*152*	*19(1.2)*	*0.041*	*317*	*39.7(5.1)*	*0.427*
	*Apr-16*	*150*	*18.8(2.2)*	*0.000*	*262*	*32.8(3)*	*0.161*
	*May-16*	*60*	*7.5(2)*	*0.000*	*121*	*15.1(3)*	*0.000*
	Sep-16	40	5(1.2)	0.000	84	10.5(1.9)	0.000
	Nov-16	38	4.8(1.1)	0.000	69	8.6(1.2)	0.000
	*Jan-17*	*174*	*21.8(3)*	*N/A*	*283*	*35.4(3.4)*	*N/A*
	Subtotal	614			1136		
TRI	*Jan-16*	*3*	*0.4(0.2)*	*0.000*	*28*	*3.5(0.7)*	*0.000*
	*Apr-16*	*7*	*0.9(0.4)*	*0.000*	*18*	*2.3(1.1)*	*0.000*
	Aug-16	8	1(0.3)	0.000	17	2.1(0.4)	0.000
	Oct-16	5	0.6(0.1)	0.000	13	1.6(0.3)	0.000
	Nov-16	3	0.4(0.1)	0.000	12	1.5(0.4)	0.000
	*Mar-17*	*6*	*1(0.6)*	*0.000*	*21*	*3.5(1.3)*	*0.000*
	*Apr-17*	*3*	*0.8(0.8)*	*0.000*	*11*	*2.8(2.3)*	*0.000*
	Subtotal	35			120		
NHO	*Jan-16*	*33*	*4.1(1)*	*0.000*	*80*	*10(1.7)*	*0.000*
	*Apr-16*	*35*	*4.4(1.4)*	*0.000*	*71*	*8.9(1.1)*	*0.000*
	Jul-16	29	3.6(0.9)	0.000	32	4(0.6)	0.000
	Oct-16	16	2(0.5)	0.000	23	2.9(0.4)	0.000
	Dec-16	18	2.3(0.6)	0.000	24	3(0.2)	0.000
	*Apr-17*	*49*	*6.1(1.1)*	*0.085*	*95*	*11.9(1.2)*	*0.909*
	Subtotal	180			325		
	TOTAL	1475			2824		

Mo-Yr: month-year of collection; N: number of *Ny. darlingi* captured; HBR: Human Biting Rate; HBR represents the average bites per person per night (b/p/n) calculated from the mean of collection days within each month/12 h per day per month (2 collectors inside and outside per night); EIR: Entomological Inoculation Rate. EIR not available for SEM January 2017. Italicized lines indicate rainy season collections

### Mosquito sampling

We used the Human Landing Catch (HLC) method to collect adult mosquitoes indoors and outdoors (within five meters of the main house door) during four nights per collection event (a total of twenty-eight nights per community). We randomly chose eight houses and during each consecutive night, we captured mosquitoes from two of these houses. Every collection was conducted from 18:00 to 06:00 h, and collectors rotated every three hours to account for the effects of individual collector variation on attractiveness to mosquitoes. All mosquitoes captured were separated by hour, trap site location (indoor/outdoor), and by community, and subsequently identified using external morphology at our laboratory in Iquitos by trained personnel using standard keys [40–42]. Specimens were maintained on silica gel at 4 °C until DNA extraction.

### Parity, daily survival rate, and life expectancy

To estimate female age composition of the mosquito population, a proportion (9% or more depending on total numbers captured, except for Jan-2016 in NHO where none were assessed for parity) of females collected were dissected to determine parity rates (PR), daily survival, and age estimation per community per season. Mosquito life expectancy (longevity) in days was calculated by Davidson’s method [43]: $Age = \frac{1}{{log{\ell}}^{P}}$, where *ℓ* is the natural logarithm of the constant 2.71828 and *P* is the probability of a mosquito surviving one day (daily survival rate). *P* was calculated as: $P=\sqrt[gc]{PR}$ [44], where *PR* is the ratio between the number of parous mosquitoes to total number of females dissected, and *gc* the duration of the gonotrophic cycle (days). As in Moreno et al*.* [18], the gonotrophic cycle of 2.19 days was used for rainy season collections, and 2.43 for dry season collections [45].

### Molecular detection of sporozoites in *Ny. darlingi*

Genomic DNA was extracted from each specimen of *Ny. darlingi* using Qiagen DNAeasy blood & tissue kits (Qiagen, Hilden, Germany), and DNA quantification conducted with a Qubit 2.0 Fluorometer (ThermoFisher Scientific, Waltham, MA). Detection of *Plasmodium* infection was conducted using real-time PCR targeting the small subunit of the 18S rRNA, with a triplex TaqMan assay (Life Technologies), as previously described [46]. RT-PCR was conducted on pools of DNA of up to five mosquitoes, in equal DNA concentration, for the presence of *P. vivax* and *P. falciparum*. Specimens from positive pools were tested individually to calculate the infection rate (IR).

### Data analysis

The human biting rate (HBR) was evaluated as the average number of *Ny. darlingi* bites per collector per night. This was based on the total number of *Ny. darlingi* collected. As in Lainhart et al. [9], human biting time patterns were graphed, plotting the average proportion of *Ny. darlingi* collected per hour comparing riverine (LUP and SEM) and highway (NHO and TRI) communities; and then tested for significant differences with the nonparametric Kolmogorov–Smirnov (KS) test in GraphPad Prism version 9.2.0 (Graphpad Software, San Diego, CA). We calculated the Entomological Inoculation Rate (EIR) by multiplying the HBR by the proportion of *Ny. darlingi* that were determined to be *Plasmodium*-positive by RT-PCR. Sporozoite rates were calculated using the number of positive mosquitoes for *Plasmodium* divided by the total number of mosquitoes tested. To calculate the monthly EIR, we combined the numbers of *P. vivax* and *P. falciparum* and then multiplied the HBR by the proportion of infected specimens per month. Our rationale for combining these two *Plasmodium* species is that even though in the 1990s more *Ny. darlingi* were found to be infected by *P. falciparum* compared with *P. vivax* in Loreto [24], more recent studies have found the opposite [10, 14, 19, 47].

To test the hypothesis that the peak biting time of *Ny. darlingi* in riverine and highway communities differs, we used average proportions of *Ny. darlingi* collected per hour in the four communities and tested for significance with the KS statistical test [9].

Count data of all *Ny. darlingi* collected in 2016–2017 rainy and dry seasons, across all four collection sites, were analysed (Additional file 1) in RStudio version 1.2.5033 (R 4.0.2; R CORE TEAM). To accommodate the overdispersion in our dataset, negative binomial regression models, using forward and backward stepwise selection for variable selection, were conducted with the MASS package [48]. The glm.nb() function, was utilized with the following independent variables: season (dry/rainy), site, biting location (indoor/outdoor), 3-h time period (18:00–21:00, 21:00–24:00, 24:00–03:00, 03:00–06:00), and their interactions. A nonparametric Kruskal–Wallis analysis was also conducted on the count data for comparison to the negative binomial regression results.

Count data of the parous and nulliparous female *Ny. darlingi* in the rainy and dry seasons, across the four collection sites, were analysed (Additional file 2) in RStudio version 1.2.5033 (R 4.0.2; R CORE TEAM). We conducted negative binomial regression models as above but using the following independent variables: season (dry/rainy), site, and 6-h time period (18:00–24:00, 24:00–06:00), and their interactions. Some of the variable categories (site, indoor/outdoor, 3-h time period) were collapsed due to small sample sizes, e.g. of nulliparous females in TRI during the rainy season (see Table 2). The parity rates between community (LUP, SEM, TRI, NHO), season (rainy, dry) and community type (highway, riverine) were compared using the chi-square test with a statistical significance of *P* < 0.05 in GraphPad Prism version 9.5.1 for Windows, GraphPad Software (San Diego, California, USA).

**Table 2:** Total number, percent and number of nulliparous, parous, and gravid female *Nyssorhynchus darlingi* collected from Lupuna (LUP), Santa Emilia (SEM), El Triunfo (TRI), and Nuevo Horizonte (NHO), 2016–2017, in addition to parity rate, daily survival rate, and life expectancy

Community	Mo-Yr	Total Captured	% Total (N)	% Nulliparous (N)	% Parous (N)	% Gravid (N)	PR	Daily survival rate (*P*)	Age (days)
LUP	Feb-16	514	32.5 (167)	2.4 (4)	71.9 (120)	25.7 (43)	0.72	0.86	6.63
	Apr-16	482	41.1 (198)	3.5 (7)	28.3 (56)	68.2 (135)	0.28	0.56	1.73
	Jul-16	287	46.3 (133)	4.5 (6)	13.5 (18)	82 (109)	0.14	0.44	1.22
	Oct-16	91	97.8 (89)	5.6 (5)	23.6 (21)	70.8 (63)	0.24	0.55	1.68
	Dec-16	70	98.6 (69)	7.2 (5)	23.2 (16)	69.6 (48)	0.23	0.55	1.66
	Jan-17	445	38.4 (171)	6.4 (11)	60.2 (103)	33.3 (57)	0.60	0.79	4.32
	Subtotal	1889	43.8 (827)	4.6 (38)	40.4 (334)	55 (455)			
SEM	Feb-16	469	9.4 (44)	4.5 (2)	70.5 (31)	25 (11)	0.70	0.85	6.25
	Apr-16	412	10.4 (43)	11.6 (5)	51.2 (22)	37.2 (16)	0.51	0.74	3.27
	May-16	181	16 (29)	17.2 (5)	55.2 (16)	27.6 (8)	0.55	0.76	3.68
	Sep-16	124	16.9 (21)	19 (4)	61.9 (13)	19 (4)	0.62	0.82	5.07
	Nov-16	107	16.8 (18)	16.7 (3)	61.1 (11)	22.2 (4)	0.61	0.82	4.93
	Jan-17	457	10.3 (47)	12.8 (6)	46.8 (22)	40.4 (19)	0.47	0.71	2.88
	Subtotal	1750	11.5 (202)	12.4 (25)	56.9 (115)	30.7 (62)			
TRI	Jan-16	31	54.8 (17)	0 (0)	100 (17)	0 (0)	1.00	1.00	N/A
	Apr-16	25	80 (20)	5 (1)	60 (12)	35 (7)	0.60	0.79	4.29
	Aug-16	25	100 (25)	20 (5)	36 (9)	44 (11)	0.36	0.66	2.38
	Oct-16	18	100 (18)	22.2 (4)	38.9 (7)	38.9 (7)	0.39	0.68	2.57
	Nov-16	15	100 (15)	20 (3)	40 (6)	40 (6)	0.40	0.69	2.65
	Mar-17	27	33.3 (9)	0 (0)	77.8 (7)	22.2 (2)	0.78	0.89	8.71
	Apr-17	14	50 (7)	0 (0)	100 (7)	0 (0)	1.00	1.00	N/A
	Subtotal	155	71.6 (111)	11.7 (13)	58.6 (65)	29.7 (33)			
NHO	Jan-16	113	0 (0)	N/A (0)	N/A (0)	N/A (0)	N/A	N/A	N/A
	Apr-16	106	53.8 (57)	12.3 (7)	59.6 (34)	28.1 (16)	0.60	0.79	4.24
	Jul-16	61	100 (61)	26.2 (16)	37.7 (23)	36.1 (22)	0.38	0.67	2.49
	Oct-16	39	100 (39)	35.9 (14)	30.8 (12)	33.3 (13)	0.31	0.62	2.06
	Dec-16	42	100 (42)	23.8 (10)	38.1 (16)	38.1 (16)	0.38	0.67	2.52
	Apr-17	144	44.4 (64)	12.5 (8)	59.4 (38)	28.1 (18)	0.59	0.79	4.20
	Subtotal	505	52.1 (263)	20.9 (55)	46.8 (123)	32.3 (85)			
	TOTAL	4299	32.6 (1403)	9.3 (131)	45.4 (637)	45.3 (635)			

^*^% Total: percent of females dissected for parous status of total captured

## Results

### Mosquito capture data and entomological indices

Overall, 4,330 Anophelinae were collected during this study, of which 4,299 were identified morphologically as *Ny. darlingi.* The rainy season accounted for 3,420 individual *Ny. darlingi* specimens (79.55%), and the dry season for 879 (20.45%). Using ITS2-PCR–RFLP as in Matson et al. [49], we identified one *Nyssorhynchus benarrochi* B individual from SEM. Thirty specimens that were provisionally morphologically identified as non-*Ny. darlingi* failed to amplify, could not be identified molecularly, and were excluded from all analyses.

**Fig. 2:**
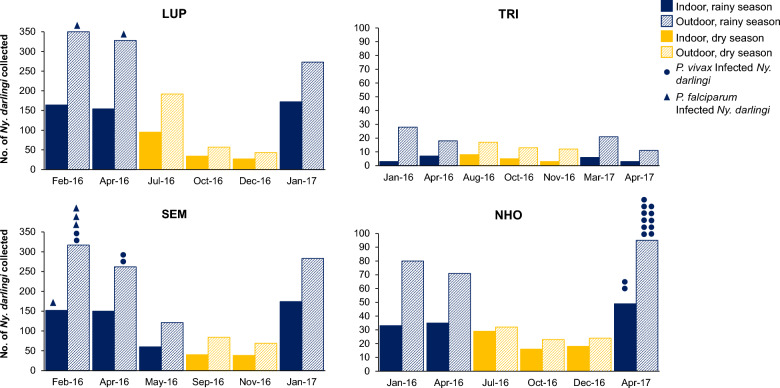
Abundance of *Ny. darlingi* collected indoors vs outdoors and rainy vs dry season in four collection sites in Loreto, Peru, 2016–2017. Collection times of infected specimens are indicated by a closed circle (*P. vivax*) or a closed triangle (*P. falciparum*)

The HBR of *Ny. darlingi* ranged from 0.4 bites per night (b/p/n) indoors in TRI (highway) to 43.8 b/p/n outdoors in LUP (riverine) (Table 1) and was generally higher during the rainy season (Table 1). The final collection of *Ny. darlingi* in January 2017 from SEM was not analysed for *Plasmodium* for logistical reasons, thus a total of 3,721 *Ny. darlingi* were tested for *Plasmodium*. Of these, 23 (0.62%) were infected. In the localities where *Plasmodium*-infected *Ny. darlingi* were detected (Fig. 2), the EIR ranged from a high of 0.909 outdoors in NHO (highway) to a low of 0.041 indoors in SEM (riverine) (Table 1). No infected *Ny. darlingi* were collected during the dry season.

### Parity, daily survival rate, and life expectancy

We dissected 1,403/4,299 or 33% of the total number of *Ny*. *darlingi* from the four localities (Table 2). There were 637 parous, 635 gravid and 131 nulliparous *Ny. darlingi* detected (Table 2 and Additional file 2: Fig. S1). The PR in the rainy season ranged from 0.28 in LUP—1.00 in TRI whereas during the dry season it was 0.14 in LUP—0.62 in SEM. In the four localities, the average estimated mosquito age was somewhat higher during the rainy season (range 1.73–8.71 days) compared with the dry season (range 1.22–5.07 days), and the highest mosquito age was detected in TRI during the rainy season (8.71 days). There was a significant difference in the proportion of parous mosquitoes by site (*χ*^2^ = 8.04, df = 3, *P* = 0.0452) as well as season (*χ*^2^ = 8.621, df = 1, *P* = 0.0033), but not for community type (*χ*^2^ = 3.724, df = 1, *P* = 0.0536).

### Seasonal, location (indoor/outdoor) and community effects

**Fig. 3:**
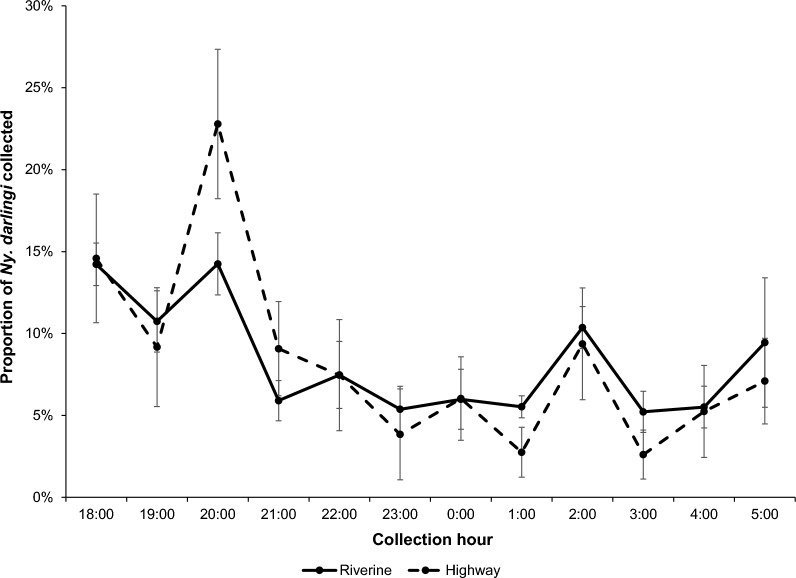
Average proportion of *Ny. darlingi* collected hourly by community type (LUP and SEM, riverine; NHO and TRI, highway). Error bars represent 95% confidence intervals

In general, more *Ny. darlingi* were captured during the rainy season compared with the dry season. Regardless of community or season, more *Ny. darlingi* were captured outdoors than indoors from all four sites; more were captured in riverine (LUP, SEM) than highway (TRI, NHO) communities (Table 1; Fig. 2). Regardless of location, season or community type, *Ny. darlingi* bit throughout the night, although there was a more pronounced early evening peak in the highway communities of TRI and NHO between 19:00 and 21:00 compared with LUP and SEM (Additional file 2: Figs. S2 and S3). There were no significant differences detected in biting patterns by the KS test (*p* = 0.8475; Fig. 3).

Results from negative binomial regression indicated significant differences in counts across sites, season, time period, and between indoor and outdoor captures (Table 3). Compared to the three other collection sites, significantly more *Ny. darlingi* were captured in LUP. In addition, more mosquitoes were collected during the rainy than dry season, outdoors versus indoors, and during the first time period (18:00–21:00) across all sites. Significant interactions (time period X indoor/outdoor, season X time period, season X site, and site X indoor/outdoor) suggest that these relationships are contextually dependent. A few of these interactions were significant only for TRI in comparison to LUP: season X site (indicating a lower rainy: dry season ratio in TRI than the other sites) and site X indoor/outdoor (indicating a higher outdoor: indoor ratio in TRI that the other sites). A Kruskal–Wallis analysis of the same *Ny. darlingi* count data supported the negative binomial regression findings (Supplemental Table 1).

**Table 3:** Negative binomial regression model of *Nyssorhynchus darlingi* counts collected in four sites (Lupuna, Nuevo Horizonte, Santa Emilia, El Triunfo) during rainy and dry seasons

Variable	*β*	*e*^*β*^	SE	*p* value
Intercept	1.71	5.60	0.11	< **0.0001**
Site (ref = Lupuna)				
NHO	− 1.13	0.32	0.14	< **0.0001**
SEM	− 0.27	0.76	0.13	**0.0372**
TRI	− 2.49	0.08	0.22	< **0.0001**
Season (ref = dry)	1.17	3.23	0.11	< **0.0001**
Time period (ref = 18:00—21:00)				
21:00–24:00	− 1.34	0.26	0.16	< **0.0001**
24:00–03:00	− 0.66	0.52	0.14	< **0.0001**
03:00–06:00	− 0.50	0.61	0.14	< **0.0001**
Indoor/outdoor (ref = indoor)	0.56	1.76	0.11	** < 0.0001**
Time period x indoor/outdoor				
21:00–24:00 outdoor	0.53	1.70	0.14	< **0.0001**
24:00–03:00 outdoor	0.00	1.00	0.14	0.9973
03:00–06:00 outdoor	− 0.14	0.87	0.14	0.3190
Season x time period				
Rainy × 21:00–24:00	0.34	1.41	0.15	**0.0248**
Rainy × 24:00–03:00	0.02	1.02	0.14	0.8997
Rainy × 03:00–06:00	− 0.42	0.66	0.14	**0.0037**
Season x site				
Rainy NHO	− 0.24	0.79	0.15	0.1049
Rainy SEM	0.05	1.05	0.13	0.7085
Rainy TRI	− 0.74	0.48	0.20	**0.0002**
Site x indoor/outdoor				
NHO outdoor	− 0.06	0.94	0.14	0.6710
SEM outdoor	− 0.01	0.99	0.12	0.9037
TRI outdoor	0.60	1.83	0.22	**0.0062**

*β*: regression coefficient; *e*^*β*^: exponentiated regression coefficient (z-value); SE: Standard error

Significance level *p* < 0.05 in bold

### Malaria incidence data

During our study (2016–2017), the Annual Parasite Index (API), based on microscopic identification of blood smears, ranged from a low of 37.8 (TRI 2016) to a high of 504.1 in LUP (Table 4). In 2017, two of the communities registered a lower API compared with 2016: TRI, population 238, increased marginally from 37.8 to 54.6 and SEM, population 204, increased from 181.4 to 279.4. In the four communities during the time-frame of the present study (2016–2017) more malaria cases (~ 65%) were *P. vivax* (~ 65%) compared with *P. falciparum* (35%)*.* These values are similar to national Peruvian averages for 2016–2017 [28, 50]*.*

**Table 4:** Number of malaria cases in the four sampled communities in Loreto, Peru (2016 and 2017)

Community	Population	No. cases 2016	API 2016	No. cases 2017	API 2017
Lupuna	365	184	504.1	163	446.6
Santa Emilia	204	37	181.4	57	279.4
El Triunfo	238	9	37.8	13	54.6
N. Horizonte	375	61	162.7	44	117.3

*N* Nuevo; Data from Ministry of Health, Iquitos, Peru; API: Annual Parasite Index calculated as number of confirmed malaria cases per 1,000 individuals

## Discussion

Results are congruent with previous studies in Amazonian Peruvian of *Ny. darlingi* that found the highest risk of acquiring malaria to be outdoors before midnight during the rainy season. Our new data underscore the low risk of local malaria transmission during the dry season, due in part to a highly significant reduction in *Ny. darlingi* abundance together with significantly lower parity rates, compared with the rainy season. In agreement with previous studies in the peri-Iquitos region of Loreto Department in Amazonian Peru [10, 19], *Ny. darlingi* was the predominant anopheline species collected in the present study (99.3%). The HBR, IR and EIR were all within the range of other similar regional studies that have focused mainly on *Ny. darlingi* (Table 1).

In peri-Iquitos, *Ny. benarrochi* B appears to be relatively uncommon, as previous reports demonstrate [14, 19, 51, 52]. In some regions, notably Datem del Marañon province, it was [8] and remains [53, 54] highly abundant. It is a regional or secondary *Plasmodium* vector in southern Colombia, eastern Peru [55], eastern (Amazonian) Ecuador [53] and Datem del Marañon [54].

Despite high net distribution and cover and access (many residents in the present study use both *tocuyos* and LLINs as we have shown previously [17, 19], *Plasmodium* continues to be transmitted and the APIs, especially in the two riverine villages of LUP and SEM, were high in both 2016 and 2017 (Table 4). In similar villages in Mazan district, Loreto region, Peru, we demonstrated that during the early evening (17:00–20:00), between 20 and ~ 80% of the human population was protected under nets, leaving adequate hosts available for mosquitoes to obtain a blood meal [19]. The most likely explanation for transmission in the present study is that some of the residents were not using their bednets during the early evening hours and therefore not protected. We observed in this study as well as in [19], that residents indoors were occupied eating or watching television, not using their nets, or they were outdoors bathing in a nearby river or playing soccer. It has been suggested that interventions that are more in synch with the malaria endemic community lifestyles and can be incorporated into routine activities will have a better chance of protecting individuals [56].

Notably, the riverine village of LUP has persisted as a malaria transmission hot spot for several years [28]. In the present study, the highest *Ny. darlingi* HBR was reported in LUP and there were significantly more *Ny. darlingi* collected in this village compared with the other three communities (Tables 1 and 3). The possible explanations for the complex malaria transmission scenario in LUP are varied: (1) the high biting rate may be an adaptation to increase *Plasmodium* transmission as infectivity rates in *Ny. darlingi* are relatively low [25]; (2) our prior larval sampling in LUP revealed that a slow-moving stream traversing the village has been a productive and permanent breeding site for *Ny. darlingi* [37]; (3) high *Plasmodium* genetic diversity and gene flow among geographically relatively far-flung communities including LUP and SEM facilitates the movement of malaria parasites [57]; (4) recurrent seasonal flooding adds to the abundance and fluctuations of *Ny. darlingi* populations; and (5) there is transmission microheterogeneity within the village of LUP [58]. It is difficult to quantify the contribution of each of these factors but integrating multiple effective interventions such as regular distribution of LLINs and/or impregnated hammocks and hammock nets, (after testing for effectiveness in reduction of indoor and outdoor transmission) to every resident; detection and monthly treatment of larval breeding sites (as in [59]); and routine detection and treatment of both symptomatic and asymptomatic/sub-microscopic *Plasmodium* carriers [60] would undoubtedly reduce malaria transmission. A comparison of human blood samples tested for presence of *Plasmodium* spp. in Santa Emilia by microscopy versus PCR by Ramirez [61] underscore the importance of testing and treatment of both symptomatic and asymptomatic/sub-microscopic *Plasmodium* carriers to cut transmission. A multi-pronged programme (PAMAFRO) was successful in dramatically reducing malaria in Amazonian Peru from 2005 to 2010 [28]. The Plan Maria Cero in Loreto, implemented from 2017 to 2020, reduced malaria cases by 75% [62]. Subsequently, in 2022, the national malaria elimination plan was launched with the main objective of reducing malaria cases in Peru by 90% by 2030 [63].

The propensity of the primary regional vector *Ny. darlingi* to bite outdoors in the early evening, at least in many riverine communities in the Amazon [64, 65] when residents are outdoors eating, relaxing, or working, constitutes a major coverage gap [19]. One plausible vector control intervention for local communities with housing that frequently includes incomplete or fewer than four walls could be the use of eave ribbons impregnated with a mosquito repellent such as those that have been shown to be highly effective in several sub-Saharan malaria endemic countries [66–68]. Clearly, such interventions would need to be tested rigorously in the Latin American context.

We were able to collect adequate sample sizes during the dry season for analysis in this study (*n* = 879 (20.45%)) and detected significant seasonal differences in abundance (Table 3) and parity. The lack of dry season *Ny. darlingi* infected with *Plasmodium* supports earlier findings that the risk for transmission in this region is during the rainy season, although farther west, in Datem del Marañon province, even in the dry season there is a risk of acquiring malaria [54]. There are two probable reasons for this. First, during the low-transmission (dry) season, despite a substantial burden of sub-microscopic infections in Loreto [12, 61] and the fact that persons with submicroscopic malaria can infect anophelines [69], generally very few mosquitoes are infected at low gametocyte densities with standard membrane feeders and transmission is considered much less likely to occur [60, 70]. Nevertheless, a comparison of microscopy versus real time PCR results of *Plasmodium*-positive residents from the community of Santa Emilia from January–September 2016, demonstrated a very biologically significant difference i.e., 6.5% (92/1416) infectivity by microscopy and 24.34% (295/1212) by PCR. The data support the consideration of testing and treating asymptomatic inhabitants, particularly of malaria hotspots.

The second reason is that malaria transmission occurs not only in villages but also in temporary locations (for example, logging or mining camps) linked to seasonal occupation [10, 12]. We did not collect anopheline specimens from such localities to test for this study but the Parker et al*.* [10] study along the Mazan River during both rainy and dry seasons detected two of 967 *Ny. darlingi* specimens infected, one positive for *P*. *falciparum* and one for *P. vivax*, from occupation-related logging sites.

The peak biting patterns for *Ny. darlingi* reported here are similar to those in Lainhart et al*.* [9], in that they differ between riverine and highway communities, albeit not significantly. Thus, in the present study, there was no significant difference between seasonal patterns, and we reject our initial hypothesis of differences between village types (riverine and highway) and instead propose that these biting patterns result from the spatiotemporal availability of human (and possibly other animal and/or bird) hosts [9, 18, 71, 72].

Our parity data indicate that the majority of *Ny. darlingi* females seeking a human bloodmeal are parous, i.e., older, as previous studies have demonstrated in Amazonian Peru [18] and Brazil [45] and, therefore, they could potentially be infected with *Plasmodium*. However, only during the March collection in TRI were samples of *Ny. darlingi* old enough (range 7.24–9.13 days) to sustain the *P. vivax* sporogonic cycle, calculated with the Moshkovsky method in Barros et al*.* [45]). Parity rates were higher for *Ny. darlingi* during peak transmission (second half of the rainy season and beginning of the dry season) in LUP, NHO and TRI, although this does not hold for SEM (Table 2). Limitations of this study include the high variability in the range of mosquitoes that were dissected and analysed to estimate parity among sites and seasons, as well as in some cases, very low numbers of nulliparous mosquitoes, such that the vector age could not be calculated. Dissections can be inaccurately interpreted and training in the apparently simple technique is essential. Determination of age has long been a vexing issue in vector biology but a promising new surveillance method, based on deep learning of mid-infrared spectra of mosquito cuticle, was able to accurately and cost-effectively identify both species and age class among three closely related Africa vectors, *Anopheles gambiae*, *Anopheles arabiensis* and *Anopheles coluzzii* [73]. Hopefully, this method can soon be applied to Latin American and other regional malaria vectors and make a significant impact on malaria transmission reduction.

## Conclusions

Malaria incidence in Peru is highest when the interactions between the various ecological and human factors are optimal for malaria transmission [28] and may be region specific. For example, in Roraima state, Brazil, peak malaria incidence and the highest parity rates occurred during the dry season [45], whereas in southern Venezuela, the peak malaria incidence occurred one month after peak biting rates by *Ny*. *darlingi* and *Nyssorhynchus marajoara*, and there was no correlation with rainfall [74]. In the present study, malaria transmission is optimal during the rainy season when there is an abundance of breeding sites, parous female *Ny*. *darlingi*, and available human hosts, especially outdoors during the early evening hours that coincide with the peak human biting rates of *Ny. darlingi.*

### Supplementary Information

**Additional file 1: Count dataset**. *Nyssorhynchus darlingi* by collection site, season (rainy/dry), biting location (indoor/outdoor), and time period for all four collection sites (LUP, NHO, SEM, TRI).

**Additional file 2: Table S1.** Kruskal-Wallis analysis on ranked abundance of *Nyssorhynchus darlingi*, in four collection sites (Lupuna, Nuevo Horizonte, Santa Emilia, El Triunfo), during rainy and dry seasons 2016-2017. **Figure S1.** Average parity rate for each collection site comparing: **A** Before vs. after midnight collections; **B** Indoor vs. outdoor collections; **C** Rainy vs. dry collections. **Figure S2.** Average proportion of *Ny. darlingi* collected hourly biting indoor vs. outdoor for each collection site. Confidence intervals not shown for clarity. **Figure S3.** Average proportion of *Ny. darlingi* collected hourly biting by season for each collection site. Confidence intervals not shown for clarity.

## Data Availability

The data supporting the conclusions of this article are included within the article. The raw data used and/or analysed in this study are available from the corresponding author upon reasonable request.
