# Supplementary material for: Effect of spatiotemporal variables on abundance, biting activity and parity of Nyssorhynchus darlingi (Diptera: Culicidae) in peri-Iquitos, Peru
Source: Malar J. 2024 Apr 19;23:112. doi: 10.1186/s12936-024-04940-z (PMC11031940; doi:10.1186/s12936-024-04940-z)
Supplement: Supplementary file 2 — Additional file 2: Table S1. Kruskal-Wallis analysis on ranked abundance of Nyssorhynchus darlingi, in four collection sites (Lupuna, Nuevo Horizonte, Santa Emilia, El Triunfo), during rainy and dry seasons 2016-2017. Figure S1. Average parity rate for each collection site comparing: A Before vs. after midnight collections; B Indoor vs. outdoor collections; C Rainy vs. dry collections. Figure S2. Average proportion of Ny. darlingi collected hourly biting indoor vs. outdoor for each collection site. Confidence intervals not shown for clarity. Figure S3. Average proportion of Ny. darlingi collected hourly biting by season for each collection site. Confidence intervals not shown for clarity. [file 12936_2024_4940_MOESM2_ESM.pdf]

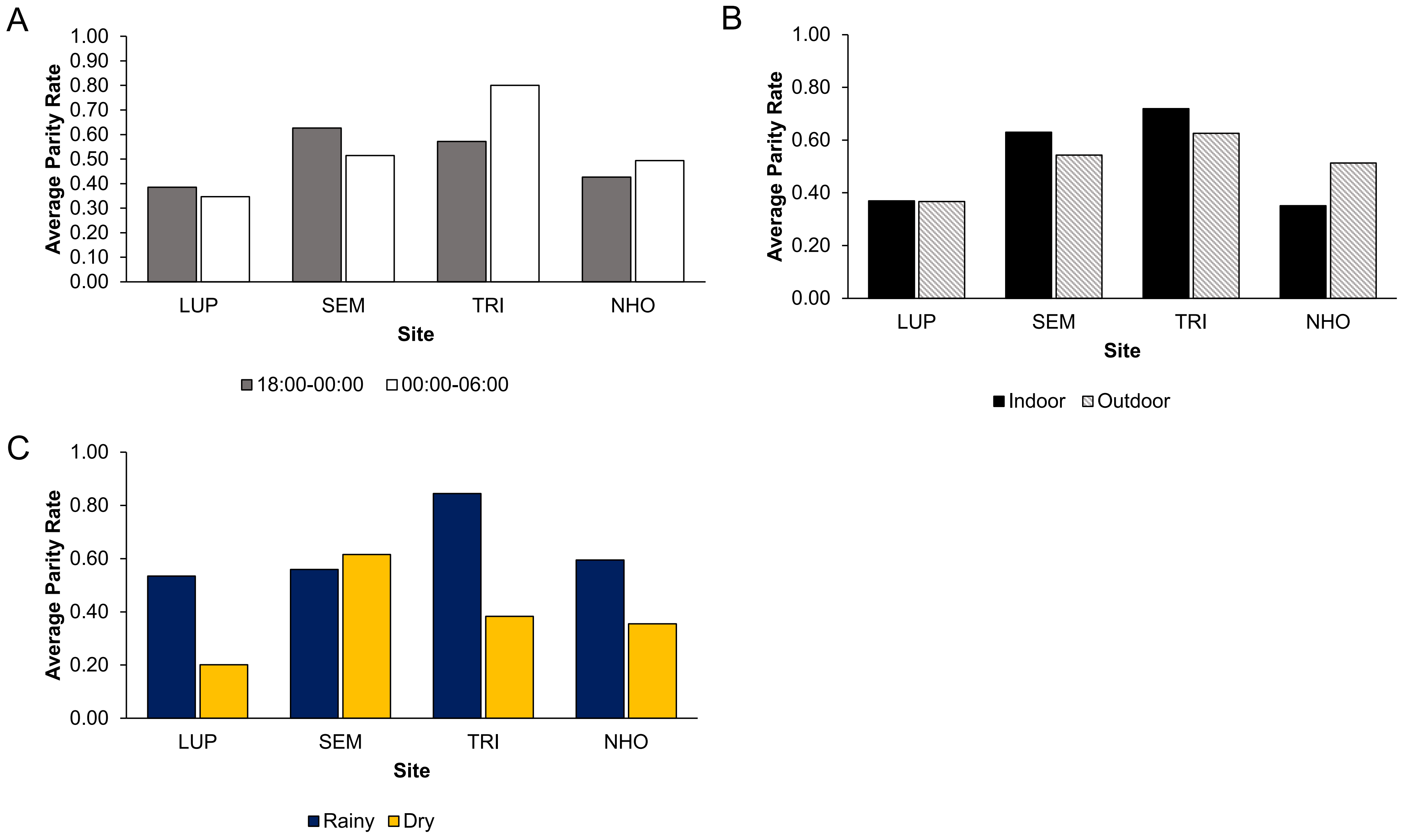

Figure S1. Average parity rate for each collection site comparing: A) Before vs. after midnight collections; B) Indoor vs. outdoor collections; C) Rainy vs. dry collections.

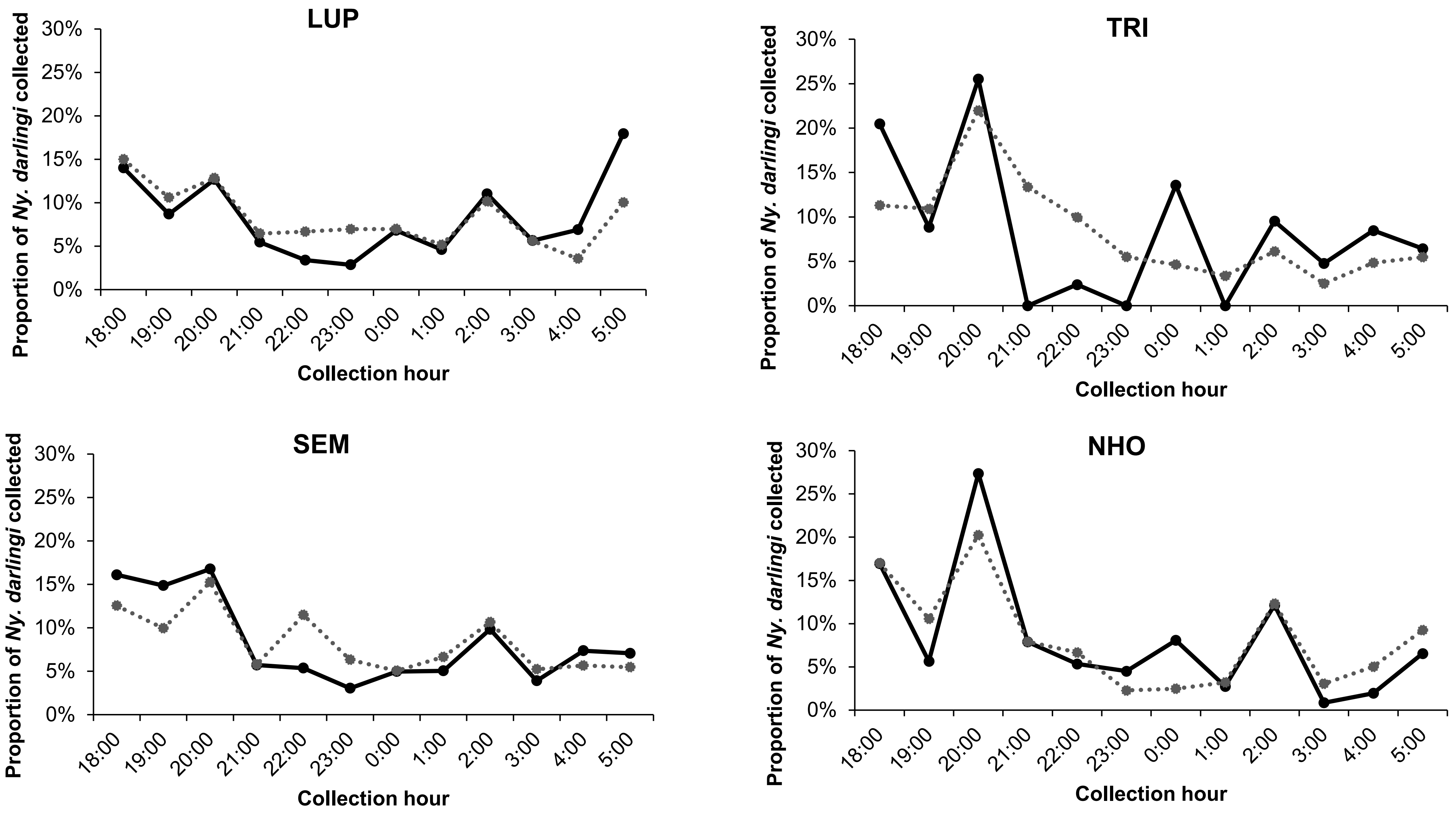

Figure S2. Average proportion of *Ny. darlingi* collected hourly, biting indoor (solid black line) vs. outdoor (gray dotted line) for each collection site. Confidence intervals not shown (for clarity).

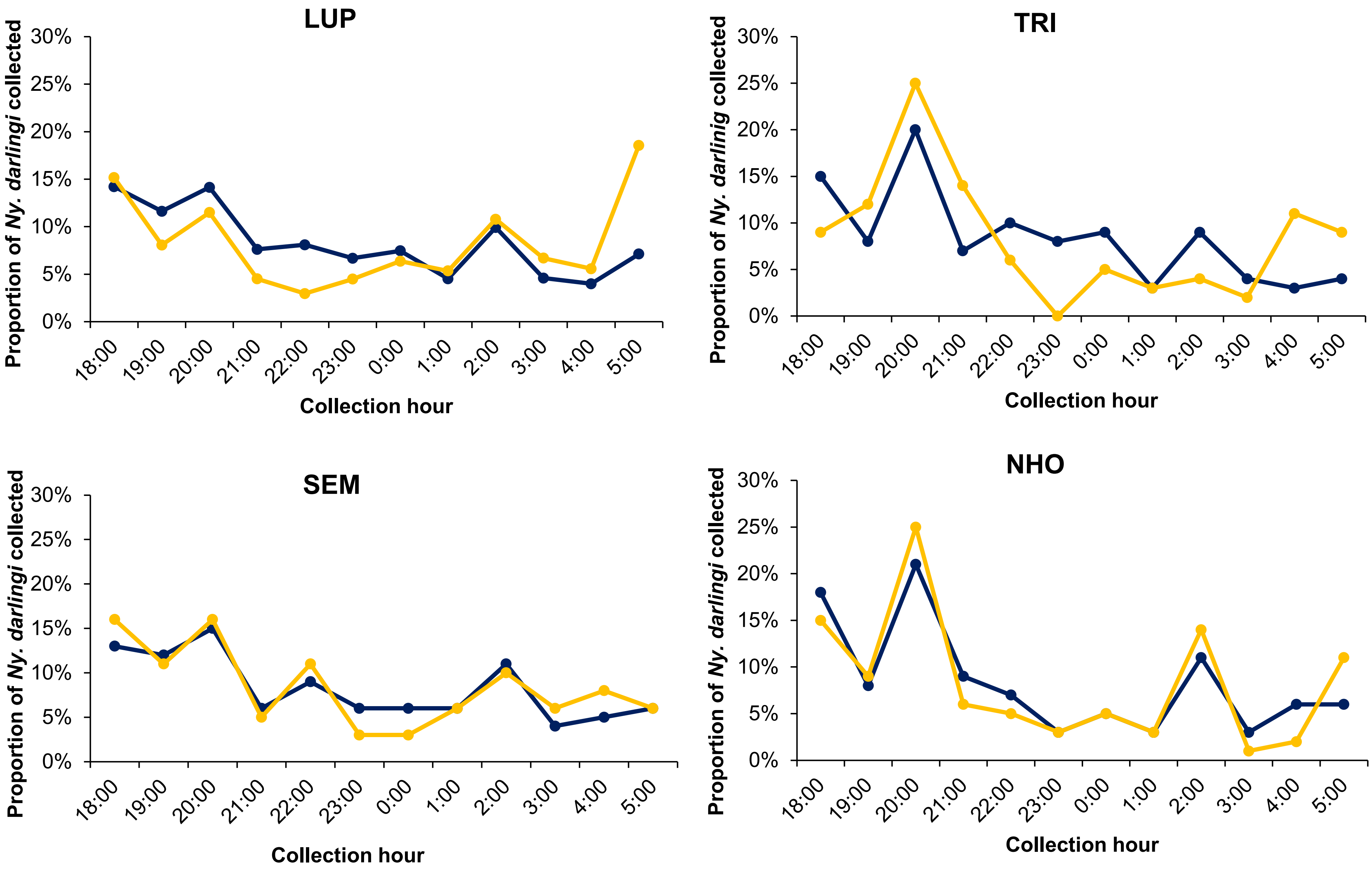

Figure S3. Average proportion of *Ny. darlingi* collected hourly, by season (blue: rainy; yellow: dry) for each collection site. Confidence intervals not shown (for clarity).

Table S1. Kruskal-Wallis analysis on ranked abundance of *Nyssorhynchus darlingi*, in four collection sites (Lupuna, Nuevo Horizonte, Santa Emilia, El Triunfo), during rainy and dry seasons 2016-2017.

Variable	Degrees of freedom	F-value	p value
Season	1	385.34	< 0.0001
Site	3	390.92	< 0.0001
Indoor/outdoor	1	145.20	< 0.0001
Time period	3	52.29	< 0.0001
Season x site	3	31.80	< 0.0001
Season x indoor/outdoor	1	3.90	0.0486
Season x time period	3	6.347	0.0003
Site x time period	9	3.685	0.0002
Indoor/outdoor x time period	3	3.66	0.0122
Season x site x indoor/outdoor	6	2.448	0.0238
Site x indoor/outdoor x time period	9	2.13	0.0253
